# Female fertility does not require *Bmal1* in suprachiasmatic nucleus neurons expressing arginine vasopressin, vasoactive intestinal peptide, or neuromedin-S

**DOI:** 10.3389/fendo.2022.956169

**Published:** 2022-08-05

**Authors:** Karen J. Tonsfeldt, Laura J. Cui, Jinkwon Lee, Thijs J. Walbeek, Liza E. Brusman, Ye Jin, Michihiro Mieda, Michael R. Gorman, Pamela L. Mellon

**Affiliations:** ^1^ Department of Obstetrics, Gynecology, and Reproductive Sciences, Center for Reproductive Science and Medicine, University of California, San Diego, La Jolla, CA, United States; ^2^ Center for Circadian Biology, University of California, San Diego, La Jolla, CA, United States; ^3^ Department of Psychology, University of California, San Diego, La Jolla, CA, United States; ^4^ Department of Integrative Neurophysiology, Graduate School of Medical Sciences, Kanazawa University, Kanazawa, Japan

**Keywords:** circadian clock, fertility, luteinizing hormone surge, arginine vasopressin, vasoactive intestinal peptide, neuromedin-s, suprachiasmatic nucleus

## Abstract

Disruptions to the circadian system alter reproductive capacity, particularly in females. Mice lacking the core circadian clock gene, *Bmal1*, are infertile and have evidence of neuroendocrine disruption including the absence of the preovulatory luteinizing hormone (LH) surge and enhanced responsiveness to exogenous kisspeptin. Here, we explore the role of *Bmal1* in suprachiasmatic nucleus (SCN) neuron populations known to project to the neuroendocrine axis. We generated four mouse lines using Cre/Lox technology to create conditional deletion of *Bmal1* in arginine vasopressin (*Bmal1^fl/fl^:Avp^cre^*), vasoactive intestinal peptide (*Bmal1^fl/fl^:Vip^cre^*), both (*Bmal1^fl/fl^:Avp^cre^+Vip^cre^*), and neuromedin-s (*Bmal1^fl/fl^:Nms^cre^*) neurons. We demonstrate that the loss of *Bmal1* in these populations has substantial effects on home-cage circadian activity and temperature rhythms. Despite this, we found that female mice from these lines demonstrated normal estrus cycles, fecundity, kisspeptin responsiveness, and inducible LH surge. We found no evidence of reproductive disruption in constant darkness. Overall, our results indicate that while conditional *Bmal1* knockout in AVP, VIP, or NMS neurons is sufficient to disrupted locomotor activity, this disruption is insufficient to recapitulate the neuroendocrine reproductive effects of the whole-body *Bmal1* knockout.

## Introduction

Ovulation is produced by a hormone surge, which is temporally gated to the end of the subjective night in women (1) and rodents (2, 3). Kiss1 neurons in the anteroventral periventricular nucleus (AVPV) release kisspeptin on to gonadotropin-releasing hormone (GnRH) neurons, which prompts the release of a bolus of GnRH. This surge of GnRH stimulates gonadotropes in the pituitary to release luteinizing hormone (LH) and follicle-stimulating hormone in a similar surge, which prompts ovulation. The temporal regulation of the LH surge is believed to arise from the suprachiasmatic nucleus (SCN).

The hypothalamic SCN governs circadian rhythms, including locomotor behavior, hormone release, feeding behavior, and other circadian processes (4). SCN neurons generate circadian rhythms through a complex molecular feedback loop, in which transcription factors CLOCK and *BMAL1* dimerize and initiate transcription of clock-controlled genes, including the clock components *Cryptochrome* (*Cry*) and *Period* (*Per*). CRY and PER dimerize and suppress their own transcription in a 24-hour oscillation (5). Several studies indicate that a direct, daily SCN signal triggers the LH surge when estradiol (E2) is sufficiently high. Pharmacological block of the central nervous system on proestrus delays the surge a full day, until the next afternoon (2, 6), and ovariectomized mice and rats with E2 replacement have daily afternoon LH surges (7, 8). Lesion of the SCN abolishes the LH surge (9, 10), which cannot be recovered by an SCN transplant (11), indicating that direct projections from the SCN are critical for fertility.

Loss of *Bmal1*, a core clock gene, results in behavioral arrhythmicity (12) and infertility (13–15). *Bmal1* knockout mice do not demonstrate an LH surge (13, 16), although these mice still ovulate irregularly through an unknown mechanism (17). Previous work has determined that defective progesterone synthesis in the ovary leads to implantation failure, contributing to the infertility of the *Bmal1* knockout female (18). However, ovaries from steroidogenic cell-specific *Bmal1* knockouts can continue to produce offspring when transplanted into a wildtype female, suggesting that progesterone production is not the only pathology underlying *Bmal1* knockout infertility (19). We previously demonstrated that *Bmal1* knockout females have a heightened LH response to exogenous kisspeptin but not GnRH, suggesting an imbalance in the hypothalamus affecting the entire axis (20).

The SCN can be divided into two main areas: a ventrolateral core that primarily expresses vasoactive intestinal peptide (VIP), and a dorsomedial shell that primarily expresses arginine vasopressin (AVP). Approximately half of SCN neurons also express Neuromedin-S (NMS), including the majority of VIP and AVP-containing neurons (21). VIP neurons in the SCN core send a direct projection to GnRH neurons (22, 23), and GnRH neurons express the VIP2R receptor (24). *In vitro*, VIP induces a moderate increase in GnRH neuron firing in a time- and E2-dependent manner (25). *In vivo*, pharmacological or genetic manipulation of VIP or its receptors can alter the timing of the LH surge and fertility (26–29). AVP neurons in the SCN shell project to AVPV Kiss1 neurons (30, 31), and Kiss1 neurons express the vasopressin receptor V1a (30). In brain slice preparations, AVP robustly stimulates Kiss1 neuron firing (32) and c-Fos induction (30), regardless of time of day or estrous cycle stage. In SCN-lesioned animals or the subfertile *Clock/Clock* mutant mouse, intracranial injection of AVP in the late afternoon rescues the LH surge through V1a signaling (33, 34). These findings indicate that either SCN projections from AVP, VIP, or both neuronal populations may act as the temporal cue to initiate the GnRH-promoted LH surge.

The goal of this study was to determine if the loss of *Bmal1* in the SCN was sufficient to recapitulate the reproductive phenotype of the whole-body *Bmal1* knockout females. We hypothesized that loss of *Bmal1* in discrete SCN populations would be sufficient to disrupt circadian rhythmicity and result in infertile females. We generated several conditional knockout animals using the Cre/Lox system designed to target the AVP-Kiss1 projection (*Bmal1^fl/fl^:Avp^cre^*) the VIP-GnRH projection (*Bmal1^fl/fl^:Vip^cre^*), both projections (*Bmal1^fl/fl^:Avp^cre^+Vip^cre^*), and further target approximately half of the SCN with neuromedin-s (*Nms^cre^*), which includes most (95%) of AVP and VIP neurons (*Bmal1^fl/fl^:Nms^cre^*) (21).

## Materials and methods

### Mice

All animal experiments were approved by the UCSD Animal Care and Use Committee. *Bmal1^fl/fl^* (35, 36) *Vip^cre^* (37), and *Nms^cre^* (21, 38) mice were obtained from Jackson Laboratories (Bar Harbor, ME). The *Avp^cre^* mouse was made as described previously (39). The *Vip^cre^* mouse was crossed with the C57-based *Bmal1^fl/fl^* for at least 4 generations (> 87.5% congenic) before experimental analysis. *Bmal1^fl/fl^:Vip^cre^* and *Bmal1^fl/fl^:Avp^cre^+Vip^cre^* mice were checked for homozygosity of *Vip^cre^* allele and only heterozygous mice were used. Mutants heterozygous for one or more cre alleles were maintained through matings of mice homozygous for *Bmal1^fl/fl^* and heterozygous for one or more cre allele, with cre-negative littermates (*Bmal1^fl/fl^*) serving as controls. Genotyping was performed using the following primers: *Bmal1^flox^* (F1: CTG GAA GTA ACT TTA TCA AAC TG, R1: GAC CAA CTT GCT AAC AAT TA, R2: CTC CTA ACT TGG TTT TTG TCT); *Avp^cre^* (F: GAG GGA CTA CCT CCT GTA CC, R: TGC CCA GAG TCA TCC TTG GC); *Vip^cre^* (F1: TCC TTG GAA CAT TCC TCA GC; F2: CCC CCT GAA CCT GAA ACA TA; R: GGA CAC AGT AAG GGC ACA CA); *Nms^cre^* (F: CCA AGT TAG CCT TCC ATA CAC C, R: AGA CGG CAA TAT GGT GGA AAA T). All mice were genotyped for germline recombination of *Bmal1*, although that was never observed in these animals. Mice were given access to food and water *ad libitum*, and were group housed in 12-hour light/12-hour dark conditions (lights on at 0600 hours and off at 1800 hours) unless otherwise stated. Light intensity in the colony inside of a cage was approximately 50 cd/m^2^ and measured using a custom board built with the lux sensor TSL2561 (Adafruit, New York).

### Home cage activity and body temperature analyses

For the behavioral studies, animals were singly housed and were provided food and water *ad libitum*. Animals underwent a brief surgery for intraperitoneal telemeter implantation (Mini-mitter, Bend, Oregon) and were allowed to recover one week. Data were collected with a VitalView data collection system (Mini Mitter, Bend, OR) in 6-minute bins. Activity was monitored for 4 weeks on 12:12 light:dark (LD), followed by two 8-hour light advances or delays, 4 weeks apart (advance and delay order varied by cohort). Two of the cohorts were given a 30 min light pulse at ZT 16 (4 hours after lights off) before being released into 24 dark (DD); the third and final cohort was released into DD without a light pulse; however, analysis was restricted to the LD and DD times due to low complete datasets due to telemeter failure. Cages were changed every 3-4 weeks, and these days were omitted from analysis. Temperature and home cage activity analyses were performed using ClockLab (Actimetrics, Wilmette IL). In some cases, only activity or temperature was collected from a given animal due to connection issues, or telemeters did not report throughout the entirety of the experiment. In these cases, data were included when available but excluded from datasets from paired analysis. X^2^ periodograms were generated for periods from 18 to 30 h using a significance criterion at 0.001. Period and periodogram amplitude were measured using the final 10 consecutive days of data within each respective paradigms; the 10 days used were the same within a cohort. Arrhythmic mice were those that did not exhibit a significant peak between these hours and were excluded from analysis.

### Fertility assays

Estrous cyclicity was determined by morning vaginal lavage for at least 21 consecutive days. After the vaginal smears were collected and dried, they were stained in methylene blue. The vaginal smears were analyzed for the presence of leukocytic cells, small nucleated cells, large nucleated cells, and anucleated cells by three independent observers (40).

### Fecundity

Fecundity was measured by pairing transgenic females (13 – 22 weeks) with males for 100 days. Because large enough cohorts were not always available at the same time, cre-positive and cre-negative litter mates were set up with WT animals at the same time in age-matched cohorts. The time to first litter, average litter size, and the total number of litters were recorded and compared between experimental pairs and the control counterparts. If an animal died during the course of the breeding study, the time to first litter and average litter size were included but total number of litters excluded.

### Kisspeptin challenges

Vaginal cytology was used to identify diestrus mice. A baseline blood sample (10 µL) was collected *via* tail tip bleed, and 2 mg/kg Kiss-10 (catalog no. 4243; Tocris Bioscience, Bristol UK) was injected intraperitoneally. Blood was collected again through the tail tip 15 min after injection. All hormone challenges were performed between ZT 4-6. The blood samples were allowed to clot at room temperature for 60 min and then were centrifuged at 2,000 x g for 15 minutes. Serum was separated and stored at -20°C until LH was measured.

### LH surge paradigm

The LH surge paradigm was modeled after the injection paradigm used in Bosch et al. (41) Female mice were bilaterally ovariectomized and were allowed to recover for 5 days. After recovery, animals were subcutaneously injected at ZT10 with 0.25 µg of β-estradiol (EB; catalog no. E8875; Sigma-Aldrich. St Louis MO) dissolved in 100 µL of sesame oil (catalog no. S3547; Sigma-Aldrich). The following day, the female mice were subcutaneously injected with 1.5 µg of EB dissolved in 100 µL of sesame oil and soiled male bedding added to the cage. The following day (7 days after ovariectomy), the female mice were either euthanized or bled at ZT12 (lights off) and blood was collected for LH analysis. A mixed cohort of surge mice were sampled the following morning at ZT2 (AM) to establish negative feedback LH levels. We used a conservative surge threshold of the average AM LH level plus 2 SD (42).

### Luteinizing hormone Milliplex assay

Singlet serum samples were run on a Milliplex analyzer (MPTMAG – 49K; MilliporeSigma, Burlington, MA) using a Luminex Magpix according to manufacturer’s instructions. Data were analyzed using Luminex software and a 5-parameter logistic curve. The LH assay had a lower detection limit of 4.8 pg/mL, a 15.2% intraassay coefficient of variation, and a 4.7% interassay coefficient of variation.

### Ovarian analysis

Age-matched cohorts of 13–26-week-old female mice were singly-housed and placed in light-tight chambers for 8 weeks to detect the effect of constant darkness conditions on ovarian function. A subset of mice had locomotor behavior monitored by passive infrared motion detectors to detect locomotor activity (Coral Plus, Visonic, Bloomfield, CT) or Mini Mitters as described above. Cages were changed every 3-4 weeks in red light. Mice were sacrificed in the morning without coordination with activity cycle because the relative histological features are long-lasting. Ovaries were dissected and placed in a mixture of 60% EtOH, 30% formaldehyde (40%), and 10% acetic acid for 24 hours, followed by immersion in 70% EtOH. Ovaries were paraffin processed by Reveal Biosciences (San Diego, CA), embedded, and sectioned at 10 µm. Haemotoxylin (Sigma-Aldrich) and eosin (Leica, Wetzlar Germany) staining was performed and the sections were imaged using an Olympus V200 Slide Scanner (UCSD Neurosciences Microscopy Core). Ovarian structures were quantified by two blinded observers and averaged, and the greatest number of structures per section for each animal was reported to avoid double-counting. Graafian follicles were determined based on the presence of an antral space.

### Statistics

Statistics were performed using GraphPad Prism 9.2 (San Diego, CA). Data are reported as mean ± standard error of mean. Specific analyses are indicated in the Results and Figure Legends with the respective data. Comparison reporting in text is formatted as *Bmal1^fl/fl^* vs mutant. Significant values were (p ≤.05) are indicated by an asterisk (*), p ≤ 0.01 by (**), p ≤ 0.001 by (***), and p ≤ 0.0001 by (***).

## Results

### *Bmal1* is required in AVP, VIP, and NMS neuron populations for normal circadian behavior

**Figure 1:**
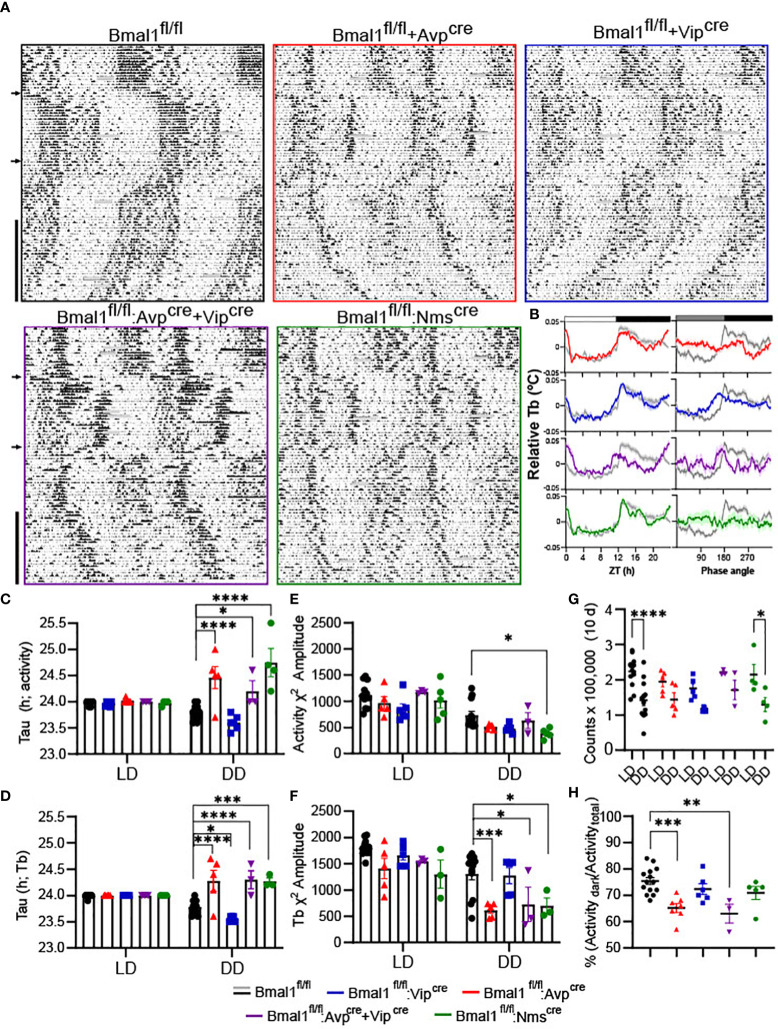
Conditional deletion of *Bmal1* disrupts home cage activity and temperature rhythms. **(A)** Representative double-plotted, normalized actograms of male *Bmal1^fl/^* ^fl^, *Bmal1^fl/fl^:Avp^cre^*, *Bmal1^fl/fl^:Vip^cre^*, *Bmal1^fl/fl^:Avp^cre^+Vip^cre^*, *Bmal1^fl/fl^:Nms^cre^* mice over a 16-week paradigm. Weeks 1-12 were spent in 12:12 LD, with 8-hour phase delays and advances occurring at week 4 and week 8 (arrows). At Week 12, animals were exposed to a 30 min light pulse (*) at ZT 20 and released into constant darkness for the remainder of the experiment (vertical black bar). Data during cage changes have been removed (gray boxes). **(B)** Body temperature (Tb) rhythms from a representative animal over 10 days in LD (left) and DD (right) compared to a representative *Bmal1^fl/fl^* (gray); data are plotted as mean ± SEM. **(C, D)** Free-running period (tau) from Chi-squared periodogram over the final 10 days of activity in the first LD period (LD) and constant darkness (DD) **(C)** or temperature **(D)** (n = 3-12). **(E, F)** Chi-squared periodogram amplitude from the same data as C and D for activity **(E)** and temperature **(F)**. Data for tau and amplitude were analyzed by 2-way ANOVA and Dunnett’s multiple comparison test. **(G)** Total activity counts over 10 days in LD vs DD for all mutants (n = 3-12). Data were analyzed by 2-way ANOVA and Sidak’s multiple comparisons test. **(H)** Nocturnality was calculated as the percentage of the activity that occurred during the dark period compared to the total activity during the day for all mutants (n = 3-14). Data were analyzed by one-way ANOVA and Dunnett’s multiple comparison test. (*) p ≤ 0.05; (**) p ≤ 0.01; (***) p ≤ 0.001; (****) p ≤ 0.0001.

To confirm behavioral effects of conditional *Bmal1* knockout, home-cage activity was quantified in male littermates (**Figure 1A**); *Bmal1^fl/fl^:Avp^cre,^Bmal1^fl/fl^:Avp^cre^+Vip^cre^*, exhibit longer free running. We observed normal spontaneous locomotor and body temperature (Tb) rhythms in mutants during LD, which were disrupted during DD (**Figure 1B**). When we analyzed ten days of data in LD or DD by Chi-squared periodogram, we found that the free running period and amplitude of the home cage activity and Tb rhythm were not significantly different in the LD condition in any genotype by 2-way ANOVA (**Table 1**; **Figures 1C–F**). In DD, *Bmal1^fl/fl^:Avp^cre^* (24.46 ± 0.21 h), *Bmal1^fl/fl^:Avp^cre^+Vip^cre^* (24.20 ± 0.20 h), and *Bmal1^fl/fl^:Nms^cre^* (24.75 ± 0.27 h) locomotor rhythms had a significantly longer free-running period than *Bmal1^fl/fl^* (23.8 ± 0.04 h) by Dunnett’s multiple comparisons test; **Figure 1C**). Similarly, we observed a significant increase in the free-running period of Tb for *Bmal1^fl/fl^:Avp^cre^* (24.28 ± 0.2 h), *Bmal1^fl/fl^:Avp^cre^+Vip^cre^* (24.30 ± 0.17 h), and *Bmal1^fl/fl^:Nms^cre^* (24.27 ± 0.07 h) compared to *Bmal1^fl/fl^* (23.78 ± 0.04 h; **Figure 1D**); additionally, we observed that *Bmal1^fl/fl^:Vip^cre^* (23.56 ± 0.02 h).had a significantly shorter period than *Bmal1^fl/fl^* (Dunnett’s multiple comparisons test).

**Table 1:** Chi squared periodogram results from telemetry measurements.

	*Bmal1^fl/fl^* (n=12)		*Bmal1^fl/fl^:Avp^cre^* (n=5)		*Bmal1^fl/fl^:Vip^cre^* (n=5-6)		*Bmal1^fl/fl^:Avp^cre^* +*Vip^cre^* (n=3)		*Bmal1^fl/fl^:Nms^cre^* (n=3-5)		2-way ANOVA Results		
	LD	LD	DD	DD	LD	DD	LD	DD	LD	DD	Interaction	Light	Genotype
Activity Tau	23.98 ± 0.011	24.02 ± 0.02	24.46 ± 0.21* †	23.8 ± 0.039	23.97± 0.02	23.6 ± 0.06 †	24.00 ± 0.00	24.20 ± 0.2 *	23.98 ± 0.03	24.75 ± 0.27 * †	**F(4,25) = 12.79,** **P < 0.001**	**F(1,25) = 7.964,** **P = 0.009**	**F(4,25) = 12.76,** **P < 0.001**
Activity amplitude	1118.93 ± 68.3	966.63 ± 115.47	509.44 ± 21.35 †	731.07 ± 74.52 †	851.68 ± 98.37	477.52 ± 31.73 †	1177.18 ± 16.07	630.36 ± 153.23 †	1015.95 ± 147.17	383.53 ± 42.04 * †	F (4, 26) = 1.012 P = 0.4194	**F (1, 26) = 85.92** **P < 0.001**	**F (4, 26) = 3.098** **P = 0.0328**
Tb Tau	23.99 ± 0.008	24.0 ± 0.0	24.28 ± 0.20*	23.78 ± 0.037 †	24.00 ± 0.0	23.56 ± 0.02 * †	24.00 ± 0.00	24.30 ± 0.17*	24.00 ± 0.00	24.27 ± 0.07 *	**F (4, 23) = 10.93** **P < 0.001**	F (1, 23) = 0.6579 P = 0.43	**F (4, 23) = 11.29** **P < 0.001**
Tb amplitude	1805.09 ± 43.62	1413.71 ± 195.04	616.0 ± 61.97 * †	1310.56 ± 118.42†	1614.71 ± 95.12	1274.24 ± 151.45 *	1546.98 ± 26.49	727.66 ± 323.71* †	1301.55 ± 269.66	698.36 ± 150.37*	F (4, 23) = 1.075 P = 0.3917	**F (1, 23) = 53.07** **P < 0.001**	**F (4, 23) = 6.397** **P < 0.001**

* Significantly different from *Bmal1^fl/fl^* in same lighting condition, Sidak’s multiple comparisons test.

†Significantly different LD condition, Sidak’s multiple comparisons test. **Bold** indicates significant effect by 2-way ANOVA.

The statistical rhyrhmicity of the detectable locomotor rhythm was significantly lower in DD only in *Bmal1^fl/fl^:Nms^cre^* (383.5 ± 42.04) compared to *Bmal1^fl/fl^* (31.1 ± 74.5; **Figure 1E**, Dunnett’s multiple comparisons test). However, the amplitude of the DD Tb rhythm was significantly lower in *Bmal1^fl/fl^:Avp^cre^* (616.0 ± 62.0), *Bmal1^fl/fl^:Avp^cre^* +*Vip^cre^* (383.5 ± 42.04), and *Bmal1^fl/fl^:Nms^cre^* (698.4 ± 150.37) compared to *Bmal1^fl/fl^* (1311.0 ± 118.4; **Figure 1F**; Dunnett’s multiple comparisons test). Additionally, the Tb rhythm of two of the five *Bmal1^fl/fl^:Nms^cre^* (40%) mice became arrhythmic in DD. An overall comparison of the locomotor and Tb rhythms found no difference in the tau calculations, whereas the amplitude of the Tb rhythms were significantly higher than the activity rhythms for *Bmal1^fl/fl^* and *Bmal1^fl/fl^:Vip^cre^* mice (2-way ANOVA, F(1, 23) = 18.48, P = 0.0003).

Total activity during the 10-day analysis periods was not different among genotypes, but was significantly different between LD and DD in *Bmal1^fl/fl^* and *Bmal1^fl/fl^:Nms^cre^* mice [**Figure 1G**; 2-way ANOVA, effect of light F(1,24) = 37.64, P<0.00001; Sidak’s multiple comparisons test]. Both the *Bmal1^fl/fl^:Avp^cre^* (65.1 ± 1.7%) and *Bmal1^fl/fl^:Avp^cre^+Vip^cre^* (63.0 ± 3.6%) exhibited significantly lower consolidation of activity in the dark period compared to *Bmal1^fl/fl^* (75.4 ± 1.3%; **Figure 1H**; one-way ANOVA). Overall, our observations of locomotor activity are similar to those previously reported (21, 43), and consistent with loss of *Bmal1* in these discrete populations producing a circadian phenotype particularly in DD. Furthermore, data from passive infrared and Mini Mitters from mutant females in DD were consistent with the male data from the respective lines (data not shown).

### Conditional loss of *Bmal1* modestly increases time in diestrus in *Bmal1^fl/fl^:Nms^cre^* mice

**Figure 2:**
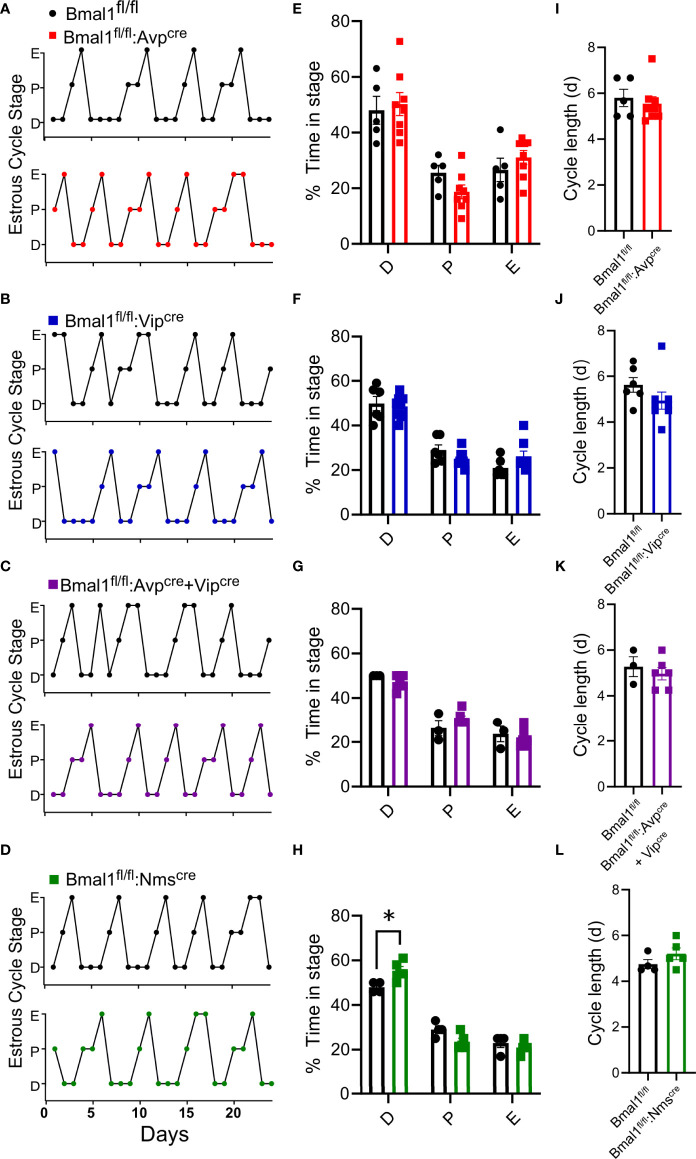
Loss of *Bmal1* produces modest effects on estrous cyclicity in *Bmal1^fl/fl^:Nms^cre^*, but not other mutants. **(A-D)** Representative cycles over 24 days from *Bmal1^fl/fl^:Avp^cre^*, *Bmal1^fl/fl^:Vip^cre^*, *Bmal1^fl/fl^:Avp^cre^+Vip^cre^*, *Bmal1^fl/fl^:Nms^cre^*, with respective cohort *Bmal1^fl/^* ^fl^ control mice. Percentage time in each estrous stage, average cycle length for completed estrous cycles, and representative cycling graphs from in **(E)** *Bmal1^fl/fl^:Avp^cre^* (n = 5-8), **(F)** *Bmal1^fl/fl^:Vip^cre^* (n = 6-8) **(G)** *Bmal1^fl/fl^:Avp^cre^+Vip^cre^* (n = 3-6), and **(H)** *Bmal1^fl/fl^:Nms^cre^* (n = 4-5), analyzed by 2-way ANOVA and Sidak’s *post hoc* test. **(I–L)** Estrous cycle length (estrous to estrous) for each line, not significant by Student’s t-test. Significant values were (p ≤.05) are indicated by an asterisk (*).

The estrus cycle was measured by daily vaginal lavage and cytology for 21-24 consecutive days. Representative staging from control and mutant mice is shown for each line (**Figures 2A–D**). The amount of time spent in each estrous stage was quantified (**Figures 2E–H**) by cohort; all lines spent significantly more time in diestrus than proestrus or estrus, but there was no effect of genotype by 2-way ANOVA [*Bmal1^fl/fl^:Avp^cre^*: F(2,33) = 30.04, P<0.0001; n = 5-8; *Bmal1^fl/fl^:Vip^cre^*: F(2,36) = 84.45, P<0.001; n = 6-8; *Bmal1^fl/fl^:Avp^cre^+Vip^cre^* (F(2,21) = 90.99 P<0.0001; n = 3-6); *Bmal1^fl/fl^:Nms^cre^* (F(2,21) =195.2, P<0.0001)]. There was a significant interaction between stage and genotype in *Bmal1^fl/fl^:Nms^cre^* [interaction F(2,21) = 8.326, P = 0.002], where *Bmal1^fl/fl^:Nms^cre^* mutant mice spend significantly longer in diestrus (**Figure 2H**; p = 0.012, Sidak’s multiple comparison’s test). Cycle length was quantified as the days between estrus, and was not significantly different between controls and all mutants by t-test (**Figures 2I–L**; *Bmal1^fl/fl^:Avp^cre^*: 5.8 ± 0.4 vs 5.5 ± 0.3, n = 5-8, p = 0.6; *Bmal1^fl/fl^:Vip^cre^*: 5.68 ± 0.3 vs 4.9 ± 0.4, n = 6-8, p = 0.2; *Bmal1^fl/fl^:Avp^cre^+Vip^cre^*: 5.3 ± 0.4 vs 5.0 ± 0.3, n = 3-6, p = 0.6; *Bmal1^fl/fl^:Nms^cre^*: 4.8 ± 0.2 vs 5.2 ± 0.3, n = 4-5, p = 0.2).

### Conditional knockout females have normal fertility

**Figure 3:**
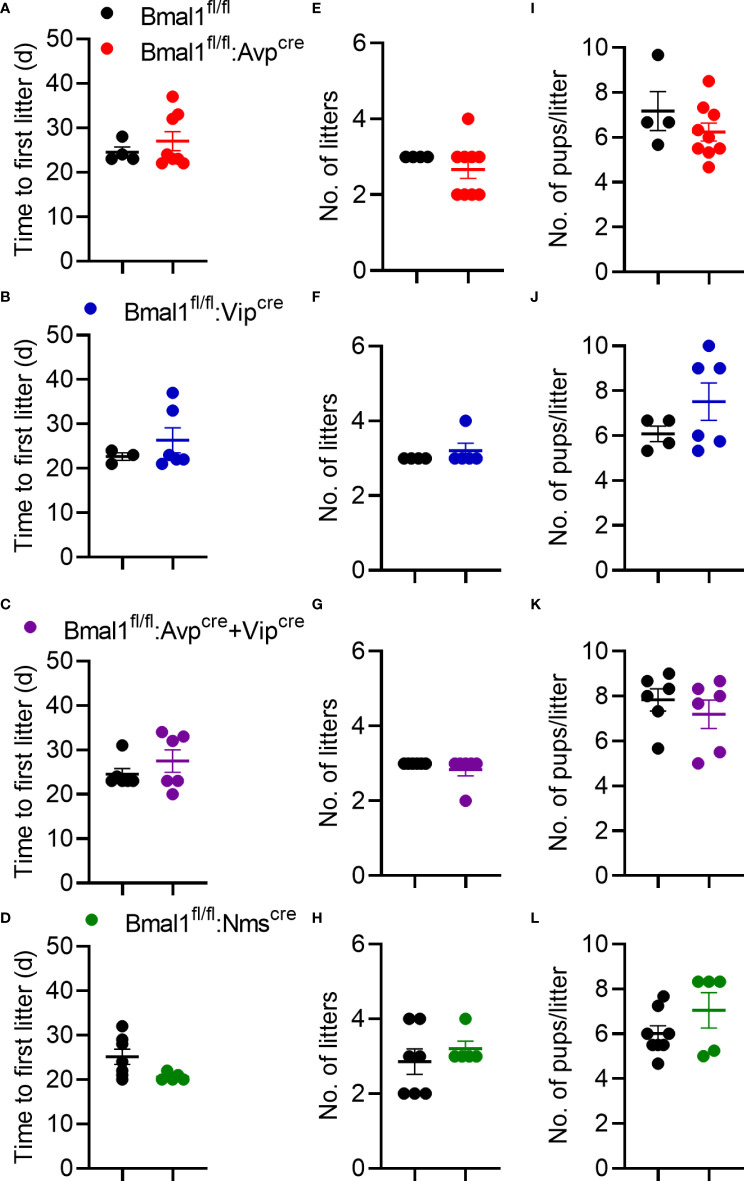
Fecundity is unaffected by conditional *Bmal1* knockout. Time to first litter **(A–D)**, number of litters in 100 days **(E–H)**, and number of pups per litter **(I-L)** were unaffected for *Bmal1^fl/fl^:Avp^cre^* (n = 4-9), *Bmal1^fl/fl^:Vip^cre^* (n = 3-6), *Bmal1^fl/fl^:Avp^cre^+Vip^cre^* (n = 6), and *Bmal1^fl/fl^:Nms^cre^* (n = 5-7) mice. Data were analyzed by unpaired t-test.

To determine if the mutant females had changes in fertility, we quantified the time to first litter, number of litters in 100 days, and size of litters. There was no significant difference in the time to first litter in any genotype compared to control (**Figures 3A–D**), although there was a trend towards fewer days to first litter in *Bmal1^fl/fl^:Nms^cre^* mice (**Figure 3D**, t-test; *Bmal1^fl/fl^:Avp^cre^*: 24.5 ± 1.1 vs 27.0 ± 2.1 days, n = 4-8, p = 0.4; *Bmal1^fl/fl^:Vip^cre^*: 22.7 ± 0.9 vs 22.3 ± 2.8 days, n = 3-6, p = 0.4; *Bmal1^fl/fl^:Avp^cre^+Vip^cre^*: 24.5 ± 1.3 vs 27.5 ± 2.5 days, n = 6, p = 0.3; *Bmal1^fl/fl^:Nms^cre^*: 25.14 ± 1.7 vs 20.6 ± 0.4 days, n = 5-7, p = 0.06). The number of litters in 100 days was not different among groups (**Figures 3E–H**, t-test; *Bmal1^fl/fl^:Avp^cre^*: 3.0 ± 0.0 vs 2.7 ± 0.2 litters, n = 4-9, p = 0.4; *Bmal1^fl/fl^:Vip^cre^*: 3.0 ± 0.0 vs 3.2 ± 0.2 litters, n = 4-5, p = 0.4; *Bmal1^fl/fl^:Avp^cre^+Vip^cre^*: 3.0 ± 0.0 vs 2.8 ± 0.2 litters, n = 6, p = 0.3; *Bmal1^fl/fl^:Nms^cre^*: 2.9 ± 0.3 vs 3.2 ± 0.2 litters, n = 5-7, p = 0.4). The number of pups per litter was also not different among the lines (**Figures 3I–L**, t-test; *Bmal1^fl/fl^:Avp^cre^*: 7.2 ± 0.9 vs 6.2 ± 0.4 pups, n = 4-9, p = 0.3; *Bmal1^fl/fl^:Vip^cre^*: 6.1 ± 0.3 vs 7.5 ± 0.8 pups, n = 4-6, p = 0.2; *Bmal1^fl/fl^:Avp^cre^+Vip^cre^*: 7.8 ± 0.5 vs 7.2 ± 0.6 pups, n = 6, p = 0.4; *Bmal1^fl/fl^:Nms^cre^*: 6.0 ± 0.4 vs 7.0 ± 0.8 pups, n = 5-8, p = 0.2). Overall, we observed no overt defects in reproductive capacity of these mice when housed on LD.

### Kisspeptin-induced LH release is unaffected by conditional loss of *Bmal1* in SCN neuron populations

**Figure 4:**
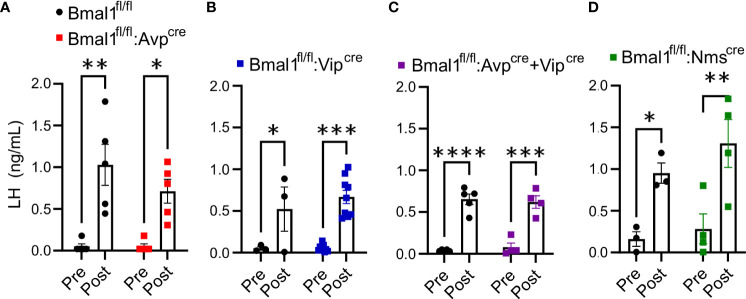
Exogenous kisspeptin produces an increase in circulating LH in conditional Bmal1 knockout mice comparable to controls. 2 mg/kg kiss-10 significantly increased LH compared to control 10 min after administration in **(A)** *Bmal1^fl/fl^:Avp^cre^* (n = 5), **(B)** *Bmal1^fl/fl^:Vip^cre^* (n = 3-9), **(C)** *Bmal1^fl/fl^:Avp^cre^+Vip^cre^* (n = 4-5), and **(D)** *Bmal1^fl/fl^:Nms^cre^* (n = 3-4) mice. Data were analyzed by two-way ANOVA and Sidak’s multiple comparison test. (*) p ≤ 0.05; (**) p ≤ 0.01; (***) p ≤ 0.001; (****) p ≤ 0.0001.

The global *Bmal1* knockout mice have an enhanced LH response to an exogenous kisspeptin challenge (20). This effect was not observed in mice selectively lacking *Bmal1* in GnRH or Kiss1 neurons (*Bmal1^fl/fl^:GnRH^cre^* or *Bmal1^fl/fl^:Kiss^cre^*), so we sought to determine if it was due to a neuroendocrine defect arising from the loss of *Bmal1* in the SCN. LH samples were collected prior to and ten minutes after intraperitoneal injection of 2 mg/kg kiss-10. All mutant and respective control mice had a significant increase in LH release in response to kisspeptin administration [**Figures 4A–D**, by 2-way ANOVA, *Bmal1^fl/fl^:Avp^cre^*: F(1,8) = 30.25, P<0.001; n = 5; *Bmal1^fl/fl^:Vip^cre^*: F(1,10) = 28.53, P<0.001; n = 6; *Bmal1^fl/fl^:Avp^cre^+Vip^cre^*: F(1,7) = 127.1 P<0.0001; n = 4-5; *Bmal1^fl/fl^:Nms^cre^*: F(1,5) = 35.34, P=0.0019; n = 3-4]. We detected no difference between mutant genotypes and controls by 2-way ANOVA [*Bmal1^fl/fl^:Avp^cre^*: F(1,8) = 1.3, P=0.28; *Bmal1^fl/fl^:Vip^cre^*: F(1,10) = 0.57, P=0.47; *Bmal1^fl/fl^:Avp^cre^+Vip^cre^*: F(1,7) = 0.003 P=0.96; *Bmal1^fl/fl^:Nms^cre^*: F(1,5) = 0.89, P=0.39)].

### Loss of *Bmal1* in SCN subpopulations does not affect the ability to mount an induced LH surge

**Figure 5:**
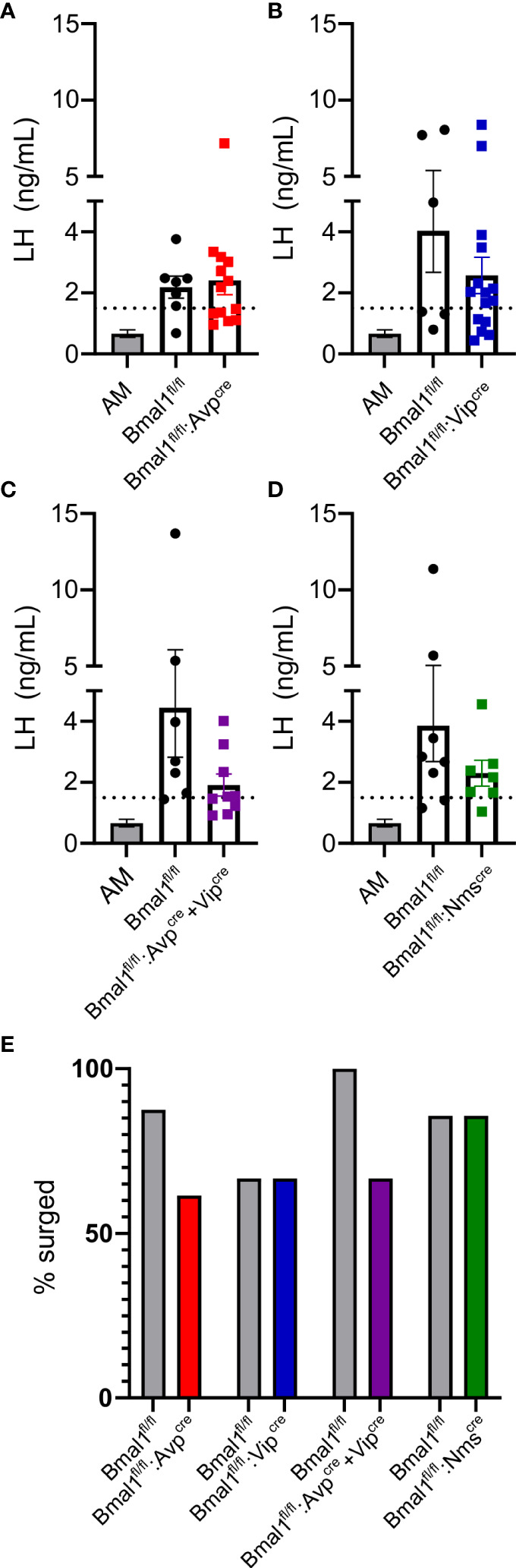
*Bmal1* mutants demonstrate an appropriately timed LH surge. LH levels at ZT12 for **(A)** *Bmal1^fl/fl^:Avp^cre^* (n = 7-12), **(B)** *Bmal1^fl/fl^:Vip^cre^* (n = 6-15), **(C)** *Bmal1^fl/fl^:Avp^cre^+Vip^cre^* (n = 7-9), and **(D)** *Bmal1^fl/fl^:Nms^cre^* (n = 7-8) mice. AM levels from mice across multiple genotypes and cohorts are plotted for comparison (same in each graph); dashed line represents surge cutoff at two standard deviations above the AM values. For all cohorts, ZT12 LH levels were significantly greater than AM levels, and there was no difference between the control and mutant populations (one-way ANOVA, Tukey’s multiple comparison’s test). **(E)** Percentage of animals above surge cutoff in each genotype (not significant by Fisher’s exact test).

To determine if *Bmal1* in these SCN subpopulations are necessary to drive an LH surge in mutant females, we used a validated OVX+E2 paradigm to promote a PM LH surge. With this model, LH is low in the morning and surges in the evening around the time of lights off. Blood was taken at ZT4 randomly across cohorts from *Bmal1^fl/fl^* and mutant animals to use as a representative sample of negative feedback (“AM”). The average LH level at ZT4 was 0.62 ± 0.38 ng/ml, resulting in a surge threshold of 1.38 ng/ml. We found that evening LH levels are not significantly different between mutants and their respective control mice (**Figures 5A–D**, t-test; *Bmal1^fl/fl^:Avp^cre^*: 2.2 ± 0.4 vs 2.4 ± 0.5 ng/ml, p = 0.75, n = 7-13; *Bmal1^fl/fl^:Vip^cre^*: 4.0 ± 1.7 vs 2.6 ± 0.6 p = 0.26 ng/ml, n = 6-15; *Bmal1^fl/fl^:Avp^cre^+Vip^cre^*: 4.4 ± 1.6 vs 1.9 ± 0.4 ng/ml, p = 0.11, n = 7-9; *Bmal1^fl/fl^:Nms^cre^*: 3.8 ± 1.2 vs 2.3 ± 0.4 ng/ml, p = 0.26, n = 7-8). Using our surge criteria, we found that the majority of animals in each group were able to mount an LH surge at the time of lights off. The percentage of animals that reached the surge threshold was not significantly different from control in any group (**Figure 5E**, Fisher’s exact test; *Bmal1^fl/fl^:Avp^cre^*: 87.5 vs 61.5%, p = 0.34; *Bmal1^fl/fl^:Vip^cre^*: 66.67 vs 66.67% p > 0.99; *Bmal1^fl/fl^:Avp^cre^+Vip^cre^* 100 vs 66.67%, p = 0.21; *Bmal1^fl/fl^:Nms^cre^*: 85.71 vs 87.5%, p > 0.99).

### Constant darkness does not alter ovarian structures in conditional mutant mice

**Figure 6:**
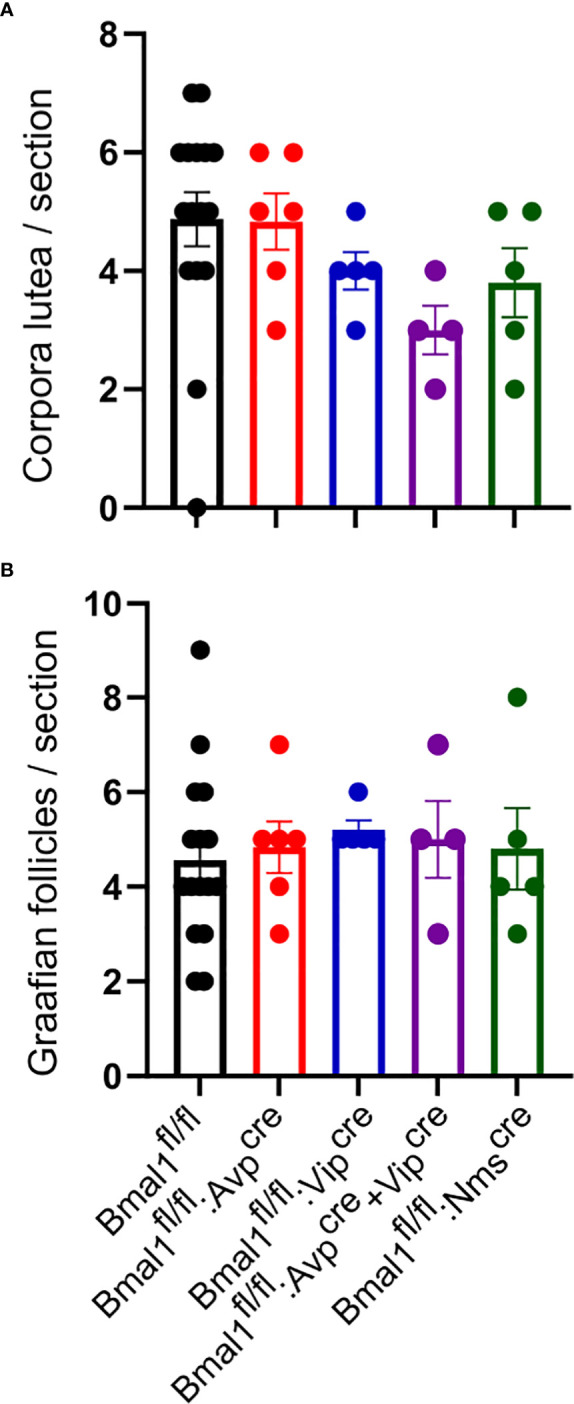
Exposure to constant darkness does not alter ovarian morphology in conditional *Bmal1* mutants. Ovarian histology was performed after six weeks in constant darkness conditions for *Bmal1^fl/fl^* (n = 16), *Bmal1^fl/fl^:Avp^cre^* (n = 6), *Bmal1^fl/fl^:Vip^cre^* (n = 5), *Bmal1^fl/fl^:Avp^cre^+Vip^cre^* (n = 4), and *Bmal1^fl/fl^:Nms^cre^* (n = 4) mice. Corpora lutea **(A, B)** Graafian follicles in the ovaries of conditional mutants compared to controls. No significant difference was found between genotypes of either structure (one-way ANOVA).

To determine if light entrainment by the SCN was sufficient to overcome a disruption in the SCN contribution to ovulation, we next determined if ovulation was still taking place after prolonged exposure to DD. We placed the mice in DD for eight weeks and examined the ovaries for evidence of ovulation and folliculogenesis by counting corpora luteal and Graafian follicles, respectively. We found no difference between the conditional mutants and *Bmal1^fl/fl^* in the number of corpora lutea (**Figure 6A**; p = 1.65, one-way ANOVA; *Bmal1^fl/fl^*: 4.9 ± 0.5 CL, n = 16, *Bmal1^fl/fl^:Avp^cre^*: 4.8 ± 0.5 CL, n = 6; *Bmal1^fl/fl^:Vip^cre^*: 4.0 ± 0.3 CL, n = 5; *Bmal1^fl/fl^:Avp^cre^+Vip^cre^* 3.0 ± 0.4 CL, n = 4; *Bmal1^fl/fl^:Nms^cre^*: 3.8 ± 0.6 CL, n = 5) or Graafian follicles (**Figure 6B**, p = 0.95, one-way ANOVA; *Bmal1^fl/fl^*: 5.6 ± 0.5 GF, n = 16, *Bmal1^fl/fl^:Avp^cre^*: 7.5 ± 0.8 GF, n = 6; *Bmal1^fl/fl^:Vip^cre^*: 4.2 ± 0.7 CL, n = 5; *Bmal1^fl/fl^:Avp^cre^+Vip^cre^* 5.2 ± 0.9 CL, n = 4; *Bmal1^fl/fl^:Nms^cre^*: 3.8 ± 1.0 CL, n = 5), indicating that ovulation progressed normally in constant darkness conditions.

## Discussion

While the conditional *Bmal1* mutant mice demonstrated robust circadian disruptions in activity and Tb, we found no evidence of female subfertility, disruption of the LH surge, or abnormal ovulation in DD conditions. We did detect mild alterations to the estrous cycle in the *Bmal1^fl/fl^:Nms^cre^* mice, but these differences were insufficient to alter fecundity. Overall, these findings were unexpected, based on the number of demonstrations of attenuated reproductive capacity following various genetic and environmental manipulations targeting the circadian system (16, 17, 44–47). The persistence of normal fertility may be due to compensatory timing mechanisms (e.g., molecular clocks in Kiss1 or GnRH neurons), the organizational effects of the light-dark cycle (particularly on peptide release) and heirarchical SCN signaling, or insufficient *Bmal1* knockout. Alternatively, the effects observed in prior reports may not generalize to circadian disruption per se.

*Clock* mutant mice and the *Bmal1* knockout are infertile and do not show an appropriately-timed LH surge in LD (17, 46). In this study, we tried to localize the effect of circadian clock disruption on fertility to discrete SCN populations by targeting VIP neurons, which project to GnRH neurons, and AVP neurons, which project to kisspeptin neurons. We also generated two models which target both AVP and VIP neurons by combining the *Avp^cre^* and *Vip^cre^* alleles, and by using the newer *Nms^cre^* allele that targets ~95% of AVP and VIP neurons as well as other SCN populations (21). We found that the LH surge can be induced at the appropriate time in these models, suggesting that the timing of the LH surge does not depend solely on *Bmal1* in AVP, VIP, or NMS neurons. The persistence of fertility in the *Bmal1^fl/fl^:Avp^cre^* +*Vip^cre^* and *Bmal1^fl/fl^:Nms^cre^* mice suggests that one signal (e.g., AVP-Kiss1) is not compensating for the other. However, both GnRH and Kiss1 neurons express endogenous clocks that have been implicated in reproduction (48, 49). While conditional deletion of *Bmal1* in either of these populations is not sufficient to disrupt fertility (50), timekeeping by these neurons may be sufficient to coordinate the surge with cell-intrinsic changes in excitability. While loss of peripheral clocks is sufficient to ablate other hormonal rhythms (51) or disrupt fertility (19, 52), the timing of the preovulatory surge may have redundant circadian mechanisms.

Since the discovery of Kisspeptin, the Kiss1-AVP projection has gained attention as the putative temporal signal that initiates the LH surge, supported by findings that exogenous AVP and optogenetic stimulation of SCN-derived AVP fibers in the AVPV stimulate Kiss1 firing in an estradiol-dependent manner (32, 53). The Battleboro rat, which is AVP and oxytocin deficient, is subfertile; however, these hormones are also important for uterine function (54). Mieda, et al. previously demonstrated that this *Bmal1^fl/fl^:Avp^cre^* combination virtually abolishes *Avp* mRNA in the SCN across the circadian day. Therefore, for the *Bmal1^fl/fl^:Avp^cre^* mutant, it is particularly surprising that no reproductive effects were observed by disrupting the AVP-Kiss1 projection. Bittman has previously reported that loss of *Bmal1* in AVP neurons disrupts the timing of the LH surge (55). That study used the same LH surge paradigm, but a different Avp^cre^ mouse, which may account for the different conclusions. Our single timepoint approach does not eliminate the possibility of a shifted LH surge in individual animals, or more subtle effects on the timing of the LH onset; however, Bittman also found no overt reproductive phenotype, which is similar to our findings. Critically, it appears that even if there is a mild disruption in surge onset, it has no profound effect on reproductive capacity in these animals.

*Clock* mutants, which have disrupted estrous cyclicity in LD, have further disruptions in DD (44, 56), indicating that light entrainment may be protective for fertility. We explored the possibility that light entrainment was sufficient for the SCN to send the temporal signal initiating the LH surge by measuring ovarian function in DD. After eight weeks in DD, we quantified corpora lutea as markers of previous ovulation and Graafian follicles as markers of follicular development. Despite the strong locomotor effects of the genetic disruption, we found no evidence of aberrant ovulation or disrupted folliculogenesis. Therefore, it appears these mice continue to ovulate in DD, although presumably at disrupted times; future studies would be needed to determine how DD and circadian disruption affects LH surge onset and fertility.

Despite the presence of a locomotor phenotype, there is documented incomplete knockout of *Bmal1* in these mouse models. Two previous studies have quantified BMAL1 expression in identical mouse models to ours: Mieda, et al., reported that *Bmal1^fl/fl^:Avp* ^cre^ had BMAL1 in only 23% of *Avp^cre^*-expressing SCN neurons compared to 90% in the *Avp^cre^* control (39); similarly, Lee, et al., previously demonstrated a reduction in BMAL1 expression in *Nms^cre^* SCN neurons from >98% down to ~20% in *Bmal1^fl/fl^:Nms* ^cre^ mice (21). Using a different *Vip^cre^* allele and same *Bmal1^fl/fl^* mouse, Todd, et al., reported that BMAL1 expression in the hemizygous *Bmal1^fl/fl^*:*Vip^cre/+^* mice persisted in 70% of cre-expressing SCN neurons (compared to 100%), whereas there was a complete knockout with a homozygous *Bmal1^fl/fl^*:*Vip^cre/cre^* (57). However, with the *Vip^cre^* created by Todd, et al., *Bmal1^fl/fl^:Vip* ^cre/+^ did not produce a locomotor or body temperature phenotype, whereas the one we report here is significantly different from the *Bmal1^fl/fl^* alone. *Vip^cre/cre^* and *Avp^cre/cre^* expression alone and in the absence of flux alleles can impair peptide production, disrupt circadian rhythms, and confound interpretation (58–60). Because AVP and VIP signaling are both implicated in the LH surge, we used mice heterozygous for cre to focus on the effect of BMAL1 disruption. Others have demonstrated that fertility is maintained in animals with >95% reduction in *Kiss1* (61) or only 10-20% of GnRH neurons (62, 63); given how robust the neuroendocrine control of reproduction is, the remaining ~20% of targeted neurons that still express *Bmal1* may be sufficient to maintain reproduction.

Overall, we demonstrate that knockdown of *Bmal1* in discrete SCN populations alters circadian rhythms but is insufficient to affect female reproduction or the ability to mount an LH surge at lights off. Further, ovulation persists even in DD, indicating that the temporal mechanism initiative ovulation is intact when the circadian clock function in demonstrably altered by *Bmal1* knockdown. Future studies are needed to determine the role of redundant circadian cues in this system and the amount of SCN function and signaling needed to maintain fertility.

## Data availability statement

The original contributions presented in the study are included in the article/supplementary material. Further inquiries can be directed to the corresponding author.

## Ethics statement

The animal study was reviewed and approved by UCSD Animal Care and Use Committee.

## Author contributions

KT, TW, MG, and PM contributed to the conception and the design of the study. KT, LC, JL, TW, and LB performed experiments. KT, LC, JL, TW, LB, and YJ analyzed data. LC wrote sections of the manuscript and KT wrote the drafts. All authors contributed the manuscript revision, read, and approved the submitted version. MM provided valuable input and the *Avp^-cre^* mouse.

## Funding

This work was supported by National Institutes of Health (NIH) Grants R01 HD072754, R01 HD100580, and R01 HD082567 (to PM). It was also supported by NIH/Eunice Kennedy Shriver National Institute of Child Health and Human Development (NICHD) P50 HD012303 as part of the National Centers for Translational Research in Reproduction and Infertility (PM). PM was also partially supported by P30 DK063491, P30 CA023100, and P42 ES010337. LB and LC were partially supported by the Doris A. Howell Research Scholarship for Women’s Health. KT was partially supported by T32 HD007203, P42 ES010337, F32 HD090837, K99 NS119291, and a UCSD Senate Grant. The UCSD Neurosciences Microscopy Core is supported by P30 NS047101.

## Acknowledgments

We would like to thank Dr. Hitoshi Okamoto for his support in supplying the *Avp^cre^* mouse. We also thank Ichiko Saotome, Jacqueline Hernandez, Jason D. Meadows, and Nathan A. Rizo for technical support, and Hanne M. Hoffman and Liz Harrison for comments on the manuscript.

## Conflict of interest

The authors declare that the research was conducted in the absence of any commercial or financial relationships that could be construed as a potential conflict of interest.

## Publisher’s note

All claims expressed in this article are solely those of the authors and do not necessarily represent those of their affiliated organizations, or those of the publisher, the editors and the reviewers. Any product that may be evaluated in this article, or claim that may be made by its manufacturer, is not guaranteed or endorsed by the publisher.

## Abbreviations

AVP, arginine vasopressin; E, estrus; P, proestrus; D, diestrus; d, days; VIP, vasoactive intestinal peptide; SCN, suprachiasmatic nucleus; NMS, neuromedin-S; LH, luteinizing hormone; GnRH, gonadotropin releasing hormone; Kiss1, kisspeptin; CRY, cryptochrome; PER, period; BMAL1, brain-muscle arntl-like factor 1; AVPV, anteroventral periventricular nucleus; LD, Light dark; DD, constant darkness; WT: Wild-type.

## References

[1] Cahill DJ Wardle PG Harlow CR Hull MG. Onset of the preovulatory luteinizing hormone surge: diurnal timing and critical follicular prerequisites. Fertil Steril (1998) 70(1):56–9. doi: 10.1016/S0015-0282(98)00113-7 9660421

[2] Everett JW Sawyer CH. A 24-hour periodicity in the "LH-release apparatus" of female rats, disclosed by barbiturate sedation. Endocrinology (1950) 47(3):198–218. doi: 10.1210/endo-47-3-198 14793479

[3] Legan SJ Karsch FJ. A daily signal for the LH surge in the rat. Endocrinology (1975) 96(1):57–62. doi: 10.1210/endo-96-1-57 1167356

[4] Abrahamson EE Moore RY. Suprachiasmatic nucleus in the mouse: retinal innervation, intrinsic organization and efferent projections. Brain Res (2001) 916(1-2):172–91. doi: 10.1016/S0006-8993(01)02890-6 11597605

[5] Welsh DK Takahashi JS Kay SA. Suprachiasmatic nucleus: cell autonomy and network properties. Annu Rev Physiol (2010) 72:551–77. doi: 10.1146/annurev-physiol-021909-135919 PMC3758475 20148688

[6] Siegel HI Bast JD Greenwald GS. The effects of phenobarbital and gonadal steroids on periovulatory serum levels of luteinizing hormone and follicle-stimulating hormone in the hamster. Endocrinology (1976) 98(1):48–55. doi: 10.1210/endo-98-1-48 942911

[7] Christian CA Mobley JL Moenter SM. Diurnal and estradiol-dependent changes in gonadotropin-releasing hormone neuron firing activity. Proc Natl Acad Sci U S A (2005) 102(43):15682–7. doi: 10.1073/pnas.0504270102 PMC1257388 16230634

[8] Legan SJ Coon GA Karsch FJ. Role of estrogen as initiator of daily LH surges in the ovariectomized rat. Endocrinology (1975) 96(1):50–6. doi: 10.1210/endo-96-1-50 1109905

[9] Gray GD Soderstein P Tallentire D Davidson JM. Effects of lesions in various structures of the suprachiasmatic-preoptic region on LH regulation and sexual behavior in female rats. Neuroendocrinology (1978) 25(3):174–91. doi: 10.1159/000122739 349417

[10] Samson WK McCann SM. Effects of suprachiasmatic nucleus lesions on hypothalamic LH-releasing hormone (LHRH) content and gonadotropin secretion in the ovariectomized (OVX) female rat. Brain Res Bull (1979) 4(6):783–8. doi: 10.1016/0361-9230(79)90012-1 393365

[11] Meyer-Bernstein EL Jetton AE Matsumoto SI Markuns JF Lehman MN Bittman EL. Effects of suprachiasmatic transplants on circadian rhythms of neuroendocrine function in golden hamsters. Endocrinology (1999) 140(1):207–18. doi: 10.1210/endo.140.1.6428 9886827

[12] Bunger MK Wilsbacher LD Moran SM Clendenin C Radcliffe LA Hogenesch JB. Mop3 is an essential component of the master circadian pacemaker in mammals. Cell (2000) 103(7):1009–17. doi: 10.1016/S0092-8674(00)00205-1 PMC3779439 11163178

[13] Alvarez JD Hansen A Ord T Bebas P Chappell PE Giebultowicz JM. The circadian clock protein BMAL1 is necessary for fertility and proper testosterone production in mice. J Biol Rhythms (2008) 23(1):26–36. doi: 10.1177/0748730407311254 18258755 PMC2862364

[14] Boden MJ Kennaway DJ. Reproduction in the arrhythmic Bmal1 knockout mouse. Reprod Fertil Dev (2005) 17(supplement):126–6. doi: 10.1071/SRB05Abs297

[15] Schoeller EL Clark DD Dey S Cao NV Semaan SJ Chao LW. Bmal1 is required for normal reproductive behaviors in Male mice. Endocrinology (2016) 157(12):4914–29. doi: 10.1210/en.2016-1620 PMC5133342 27704948

[16] Boden MJ Varcoe TJ Voultsios A Kennaway DJ. Reproductive biology of female Bmal1 null mice. Reproduction (2010) 139(6):1077–90. doi: 10.1530/REP-09-0523 20200203

[17] Chu A Zhu L Blum ID Mai O Leliavski A Fahrenkrug J. Global but not gonadotrope-specific disruption of bmal1 abolishes the luteinizing hormone surge without affecting ovulation. Endocrinology (2013) 154(8):2924–35. doi: 10.1210/en.2013-1080 23736292

[18] Ratajczak CK Boehle KL Muglia LJ. Impaired steroidogenesis and implantation failure in Bmal1-/- mice. Endocrinology (2009) 150(4):1879–85. doi: 10.1210/en.2008-1021 PMC5393263 19056819

[19] Liu Y Johnson BP Shen AL Wallisser JA Krentz KJ Moran SM. Loss of BMAL1 in ovarian steroidogenic cells results in implantation failure in female mice. Proc Natl Acad Sci U S A (2014) 111(39):14295–300. doi: 10.1073/pnas.1209249111 PMC4191810 25225411

[20] Tonsfeldt KJ Schoeller EL Brusman LE Cui LJ Lee J Mellon PL. The contribution of the circadian gene Bmal1 to female fertility and the generation of the preovulatory luteinizing hormone surge. J Endocr Society (2019) 3(4):716–33. doi: 10.1210/js.2018-00228 PMC6425515 30906911

[21] Lee IT Chang AS Manandhar M Shan Y Fan J Izumo M. Neuromedin s-producing neurons act as essential pacemakers in the suprachiasmatic nucleus to couple clock neurons and dictate circadian rhythms. Neuron (2015) 85(5):1086–102. doi: 10.1016/j.neuron.2015.02.006 PMC5811223 25741729

[22] Van der Beek EM Horvath TL Wiegant VM Van den Hurk R Buijs RM. Evidence for a direct neuronal pathway from the suprachiasmatic nucleus to the gonadotropin-releasing hormone system: combined tracing and light and electron microscopic immunocytochemical studies. J Comp Neurol (1997) 384(4):569–79. doi: 10.1002/(SICI)1096-9861(19970811)384:4<569::AID-CNE6>3.0.CO;2-0 9259490

[23] Horvath TL Cela V van der Beek EM. Gender-specific apposition between vasoactive intestinal peptide-containing axons and gonadotrophin-releasing hormone-producing neurons in the rat. Brain Res (1998) 795(1-2):277–81. doi: 10.1016/S0006-8993(98)00208-X 9622650

[24] An S Irwin RP Allen CN Tsai C Herzog ED. Vasoactive intestinal polypeptide requires parallel changes in adenylate cyclase and phospholipase c to entrain circadian rhythms to a predictable phase. J Neurophysiol (2011) 105(5):2289–96. doi: 10.1152/jn.00966.2010 PMC3094187 21389307

[25] Christian CA Moenter SM. Vasoactive intestinal polypeptide can excite gonadotropin-releasing hormone neurons in a manner dependent on estradiol and gated by time of day. Endocrinology (2008) 149(6):3130–6. doi: 10.1210/en.2007-1098 PMC2408801 18326000

[26] Weick RF Stobie KM. Vasoactive intestinal peptide inhibits the steroid-induced LH surge in the ovariectomized rat. J Endocrinol (1992) 133(3):433–7. doi: 10.1677/joe.0.1330433 1613444

[27] Harney JP Scarbrough K Rosewell KL Wise PM. *In vivo* antisense antagonism of vasoactive intestinal peptide in the suprachiasmatic nuclei causes aging-like changes in the estradiol-induced luteinizing hormone and prolactin surges. Endocrinology (1996) 137(9):3696–701. doi: 10.1210/endo.137.9.8756535 8756535

[28] van der Beek EM Swarts HJ Wiegant VM. Central administration of antiserum to vasoactive intestinal peptide delays and reduces luteinizing hormone and prolactin surges in ovariectomized, estrogen-treated rats. Neuroendocrinology (1999) 69(4):227–37. doi: 10.1159/000054423 10207274

[29] Loh DH Kuljis DA Azuma L Wu Y Truong D Wang HB. Disrupted reproduction, estrous cycle, and circadian rhythms in female mice deficient in vasoactive intestinal peptide. J Biol Rhythms (2014) 29(5):355–69. doi: 10.1177/0748730414549767 PMC4353614 25252712

[30] Williams WP 3rd Jarjisian SG Mikkelsen JD Kriegsfeld LJ. Circadian control of kisspeptin and a gated GnRH response mediate the preovulatory luteinizing hormone surge. Endocrinology (2011) 152(2):595–606. doi: 10.1210/en.2010-0943 21190958 PMC3037169

[31] Vida B Deli L Hrabovszky E Kalamatianos T Caraty A Coen CW. Evidence for suprachiasmatic vasopressin neurones innervating kisspeptin neurones in the rostral periventricular area of the mouse brain: regulation by oestrogen. J Neuroendocrinol (2010) 22(9):1032–9. doi: 10.1111/j.1365-2826.2010.02045.x 20584108

[32] Piet R Fraissenon A Boehm U Herbison AE. Estrogen permits vasopressin signaling in preoptic kisspeptin neurons in the female mouse. J Neurosci (2015) 35(17):6881–92. doi: 10.1523/JNEUROSCI.4587-14.2015 PMC6605180 25926463

[33] Palm IF van der Beek EM Wiegant VM Buijs RM Kalsbeek A. Vasopressin induces a luteinizing hormone surge in ovariectomized, estradiol-treated rats with lesions of the suprachiasmatic nucleus. Neuroscience (1999) 93(2):659–66. doi: 10.1016/S0306-4522(99)00106-2 10465449

[34] Miller BH Olson SL Levine JE Turek FW Horton TH Takahashi JS. Vasopressin regulation of the proestrous luteinizing hormone surge in wild-type and clock mutant mice. Biol Reprod (2006) 75(5):778–84. doi: 10.1095/biolreprod.106.052845 16870944

[35] RRID:IMSR_JAX Available at: https://www.jax.org/strain/007668.

[36] Storch KF Paz C Signorovitch J Raviola E Pawlyk B Li T. Intrinsic circadian clock of the mammalian retina: importance for retinal processing of visual information. Cell (2007) 130(4):730–41. doi: 10.1016/j.cell.2007.06.045 PMC2040024 17719549

[37] RRID:IMSR_JAX:010908. Available at: https://wwwjaxorg/strain/010908.

[38] RRID:IMSR_JAX:027205. Available at: https://wwwjaxorg/strain/027205.

[39] Mieda M Ono D Hasegawa E Okamoto H Honma K-I Honma S. Cellular clocks in AVP neurons of the SCN are critical for interneuronal coupling regulating circadian behavior rhythm. Neuron (2015) 85(5):1103–16. doi: 10.1016/j.neuron.2015.02.005 25741730

[40] Byers SL Wiles MV Dunn SL Taft RA. Mouse estrous cycle identification tool and images. PloS One (2012) 7(4):e35538. doi: 10.1371/journal.pone.0035538 22514749 PMC3325956

[41] Bosch MA Tonsfeldt KJ Ronnekleiv OK. mRNA expression of ion channels in GnRH neurons: subtype-specific regulation by 17beta-estradiol. Mol Cell Endocrinol (2013) 367(1-2):85–97. doi: 10.1016/j.mce.2012.12.021 23305677 PMC3570747

[42] Dungan HM Gottsch ML Zeng H Gragerov A Bergmann JE Vassilatis DK. The role of kisspeptin-GPR54 signaling in the tonic regulation and surge release of gonadotropin-releasing hormone/luteinizing hormone. J Neurosci (2007) 27(44):12088–95. doi: 10.1523/JNEUROSCI.2748-07.2007 PMC6673361 17978050

[43] Shan Y Abel JH Li Y Izumo M Cox KH Jeong B. Dual-color single-cell imaging of the suprachiasmatic nucleus reveals a circadian role in network synchrony. Neuron (2020) 108(1):164–1797. doi: 10.1016/j.neuron.2020.07.012 32768389 PMC8265161

[44] Dolatshad H Campbell EA O'Hara L Maywood ES Hastings MH Johnson MH. Developmental and reproductive performance in circadian mutant mice. Hum Reprod (2006) 21(1):68–79. doi: 10.1093/humrep/dei313 16210390

[45] Summa KC Vitaterna MH Turek FW. Environmental perturbation of the circadian clock disrupts pregnancy in the mouse. PloS One (2012) 7(5):e37668. doi: 10.1371/journal.pone.0037668 22649550 PMC3359308

[46] Miller BH Olson SL Turek FW Levine JE Horton TH Takahashi JS. Circadian clock mutation disrupts estrous cyclicity and maintenance of pregnancy. Curr Biol (2004) 14(15):1367–73. doi: 10.1016/j.cub.2004.07.055 PMC3756147 15296754

[47] Endo A Watanabe T. Effects of non-24-hour days on reproductive efficacy and embryonic development in mice. Gamete Res (1989) 22(4):435–41. doi: 10.1002/mrd.1120220409 2722124

[48] Chappell PE White RS Mellon PL. Circadian gene expression regulates pulsatile gonadotropin-releasing hormone (GnRH) secretory patterns in the hypothalamic GnRH-secreting GT1-7 cell line. J Neurosci (2003) 23(35):11202–13. doi: 10.1523/JNEUROSCI.23-35-11202.2003 PMC2932475 14657179

[49] Smarr BL Gile JJ de la Iglesia HO. Oestrogen-independent circadian clock gene expression in the anteroventral periventricular nucleus in female rats: possible role as an integrator for circadian and ovarian signals timing the luteinising hormone surge. J Neuroendocrinol (2013) 25(12):1273–9. doi: 10.1111/jne.12104 PMC3954464 24028332

[50] Tonsfeldt KJ Schoeller EL Brusman LE Cui LJ Lee J Mellon PL. The contribution of the dircadian gene Bmal1 to female fertility and the generation of the preovulatory luteinizing hormone surge. J Endocr Soc (2019) 3(4):716–33. doi: 10.1210/js.2018-00228 PMC6425515 30906911

[51] Jones JR Chaturvedi S Granados-Fuentes D Herzog ED. Circadian neurons in the paraventricular nucleus entrain and sustain daily rhythms in glucocorticoids. Nat Commun (2021) 12(5763):1–15. doi: 10.1038/s41467-021-25959-9 34599158 PMC8486846

[52] Mereness AL Murphy ZC Forrestel AC Butler S Ko C Richards JS. Conditional deletion of Bmal1 in ovarian theca cells disrupts ovulation in female mice. Endocrinology (2016) 157(2):913–27. doi: 10.1210/en.2015-1645 PMC5393362 26671182

[53] Jamieson BB Bouwer GT Campbell RE Piet R. Estrous cycle plasticity in the central clock output to kisspeptin neurons: Implications for the preovulatory surge. Endocrinology (2021) 162:bqab071. doi: 10.1210/endocr/bqab071 33824970

[54] Boer K Boer GJ Swaab DF. Reproduction in brattleboro rats with diabetes insipidus. J Reprod Fertil (1981) 61(2):273–80. doi: 10.1530/jrf.0.0610273 7205775

[55] Bittman EL. Circadian function in multiple cell types is necessary for proper timing of the preovulatory LH surge. J Biol Rhythms (2019) 34(6):622–33. doi: 10.1177/0748730419873511 PMC9206841 31530063

[56] Kennaway DJ Boden MJ Voultsios A. Reproductive performance in female clock Delta19 mutant mice. Reprod Fertil Dev (2004) 16(8):801–10. doi: 10.1071/RD04023 15740704

[57] Todd WD Venner A Anaclet C Broadhurst RY De Luca R Bandaru SS. Suprachiasmatic VIP neurons are required for normal circadian rhythmicity and comprised of molecularly distinct subpopulations. Nat Commun (2020) 11(4410):1–20. doi: 10.1038/s41467-020-17197-2 32879310 PMC7468160

[58] Joye DAM Rohr KE Keller D Inda T Telega A Pancholi H. Reduced VIP expression affects circadian clock function in VIP-IRES-CRE mice (JAX 010908). J Biol Rhythms (2020) 35(4):340–52. doi: 10.1177/0748730420925573 PMC9235543 32460660

[59] Rohr KE Telega A Savaglio A Evans JA. Vasopressin regulates daily rhythms and circadian clock circuits in a manner influenced by sex. Horm Behav (2021) 127:104888. doi: 10.1016/j.yhbeh.2020.104888 33202247 PMC7855892

[60] Cheng AH Fung SW Cheng H-YM. Limitations of the avp-IRES2-Cre (JAX #023530) and vip-IRES-Cre (JAX #010908) models for chronobiological investigations. J Biol Rhythms (2019) 34(6):634–44. doi: 10.1177/0748730419871184 31452438

[61] Popa SM Moriyama RM Caligioni CS Yang JJ Cho CM Concepcion TL. Redundancy in Kiss1 expression safeguards reproduction in the mouse. Endocrinology (2013) 154(8):2784–94. doi: 10.1210/en.2013-1222 PMC3713212 23736293

[62] Herbison AE Porteous R Pape JR Mora JM Hurst PR. Gonadotropin-releasing hormone (GnRH) neuron requirements for puberty, ovulation and fertility. Endocrinology (2008) 149(2):597–604. doi: 10.1210/en.2007-1139 18006629 PMC6101186

[63] Mayer C Boehm U. Female reproductive maturation in the absence of kisspeptin/GPR54 signaling. Nat Neurosci (2011) 14(6):704–10. doi: 10.1038/nn.2818 21516099

